# Problematic Social Media Use and Health among Adolescents

**DOI:** 10.3390/ijerph18041885

**Published:** 2021-02-15

**Authors:** Leena Paakkari, Jorma Tynjälä, Henri Lahti, Kristiina Ojala, Nelli Lyyra

**Affiliations:** Faculty of Sport and Health Sciences, University of Jyväskylä, P.O. Box 35 (L), 40014 Jyväskylä, Finland; jorma.a.tynjala@jyu.fi (J.T.); henri.o.lahti@jyu.fi (H.L.); kristiina.ojala@jyu.fi (K.O.); nelli.lyyra@jyu.fi (N.L.)

**Keywords:** social media, problematic social media use, adolescents, health

## Abstract

(1) Background: The use of social media has become an integral part of adolescents’ daily lives. However, the intensive use of social media can develop into a health-threatening addiction, but unfavourable health consequences can occur even with less use. Social media user groups categorized as *no-risk*, *moderate risk* (of developing problematic behaviour), and *problematic use* were examined with reference to their prevalence, their associations with individual determinants and health, and the increased health risk between groups. (2) Methods: The Finnish nationally representative HBSC data (persons aged 11, 13, and 15, n = 3408) and descriptive and binary logistic regression analysis were applied. (3) Results: Problematic social media use (9.4%) was most common among older age groups, and among persons with moderate/low school achievement, low health literacy, and low parental monitoring. Belonging to a moderate risk group (33.5%) was most frequent among girls, and among adolescents with low/moderate parental monitoring and health literacy. All the negative health indicators systematically increased if the respondent belonged to a moderate risk or problematic use group. (4) Conclusions: The study confirmed the association between problematic social media use and negative health outcomes and highlighted the need to pay close attention to adolescents at moderate risk who exhibited negative health outcomes.

## 1. Introduction

The recently published international report on Health Behaviour in School-aged Children [1] covered the health and health behaviour of 11-, 13-, and 15-year-old adolescents in 45 countries. The findings included a figure of 7% of adolescents reporting problematic social media use. Furthermore, 35% of adolescents could be characterized as intensive electronic media users, having online contact with people almost all the time, and throughout the day [1]. One may thus agree with the notion of Griffiths and Kuss [2] that “teenagers particularly appear to have subscribed to the cultural norm of continual online networking”. Social media refers to various internet-based semi-public and public sites and services (e.g., social network sites, blogging, and video sharing), and related tools that provide spaces for user-profiles and for user-generated content, socializing, and sharing [3,4]. Undoubtedly, for today’s young people, who have grown up with instant access to social media [5], the social media offer spaces and possibilities to find friends, reduce social isolation, and gain social support [6], with opportunities also for learning, creativity, and self-fulfilment. However, if the use develops into problematic behaviour, it may bring unfavourable consequences with it. 

Research on problematic social media use, disorder, or addiction among adolescents has increased during the last decade. However, many scholars do not refer to problematic social media use as an addiction or disorder, as the phenomenon has not yet been officially classified as such, e.g., [7]. Though a growing body of research has shown a link between problematic social media use and unfavourable health, the data leading to results and conclusions have mainly been self-reported, as have the data leading to classification as a problematic user. According to Zendle and Bowden-Jones [8], given the lack of diagnostic criteria and the limited amount of high-quality longitudinal research, not much can be said about the nature of excessive social media use and its similarities with known addictions. It is for this reason that somewhat broad terms, such as problematic social media use, have been adopted.

Similarly, because there is no shared understanding of what such problematic behaviour means or how it should be measured, there are wide variations in defining the current level of problematic use in the population [9,10]. Furthermore, if intensive engagement with social media and networking is seen as the status quo, it can be hard to define when the use becomes problematic [2].

Some scholars [9] have characterized problematic social media use via components that apply to all addictions [11], while others [10] have used criteria based on the Diagnostic and Statistical Manual of Mental Disorders (DSM-V) [12]. These have been applied to internet gaming disorder, with possibilities for defining social media disorder more generally. Following this approach, the following nine components could be used to define and measure social media disorder: preoccupation (“substantial time used to think about the activity”), withdrawal, tolerance, problems in reducing/stopping (“unsuccessful attempts to stop”), giving up other activities, continuance despite problems, deception or covering up, use to escape from or relieve negative moods, and indications of risk or loss regarding relationships or opportunities [13]. These nine components formed the basis of the HBSC study referred to above, which used the Social Media Disorder Scale with nine yes/no items [10]. Respondents with six or more “yes” answers were categorized as problematic users [14]. 

Adolescents’ problematic social media use has been viewed as a complex phenomenon with several explanatory factors. Problematic use has been explained via individual-level factors (e.g., fear of missing out [15,16]), family-level factors (e.g., childhood maltreatment [17], lessened family support [18], lower family functioning [19]), and/or friend-level factors (lower levels of friend support [18]). Macro-level factors have also been proposed, including the normalization of the surveillance culture, involving online comparison, and possibilities to find out what people are doing [20]. Gender and age have often been regarded as explanatory factors for disordered social media use. Girls are more likely to report problematic social media use than boys [1,9,21,22,23,24], and older girls more likely than younger girls to report such use [1]. Boys have been found to be more likely to report disordered internet gaming [23]. Across countries, no consistency has been found regarding the association between social media use and family affluence [1]. 

The fairly extensive use of social media among adolescents has raised concerns about the consequences for adolescents’ health and wellbeing e.g., [25,26]. Most research has focused on the link between social media use (including social network site addiction) and various psychosomatic complaints or disorders. Following a longitudinal study, Vannucci and Ohannessian [27] reported that “social media patterns appear to differentially predict psychosocial adjustment during early adolescence, with high social media use being the most problematic”. Problematic social media use has been linked to a greater likelihood of depressive symptoms [5,9,22,28,29,30,31,32], anxiety [28,32], lower self-esteem [9,28,32], social isolation [29], lower life-satisfaction [18], poorer sleep quality [21,28,32], disordered eating [33], and higher body image dissatisfaction [34]. Considering the associations with the various psychosomatic complaints, it is easy to understand why assessment of social media use has been proposed as a standard element in psychiatric assessment [35]. Furthermore, problematic social media use has been associated with unfavourable behaviours such as lower levels of physical activity [19] and impaired ability to regulate daily responsibilities [28], while higher social media use has been associated with the initiation of risky behaviours (e.g., substance use and risky sexual behaviours, [36]). For their part, digital stimuli, notably the rapid shifts in attention caused by high use of social media have been associated with cognitive factors such as a momentarily shortened attention span and lowered information processing [37]. Overall, there are indications that problematic and high social media use may predict a heightened risk for an array of negative health outcomes among adolescents.

Despite a recent increase in research on social media use and health, there have been calls for more research “to protect children and young people from the negative effects social media can have on their risk-taking behaviours, mental health, and wellbeing” [6]. This underlines the utility of obtaining a nationally representative sample to determine how social media use is associated with a comprehensive set of health indicators (examined in the same sample), and whether an association with negative health indicators exists among persons adjudged to have a heightened (albeit moderate) risk for problematic use. The study by Bányai and her colleagues [9] using a nationally representative Hungarian sample is one of the few to reveal that the likelihood of poorer health (i.e., lower self-esteem and a higher level of depressive symptoms) is already increased among adolescents with a heightened risk of problematic social media use, as compared to those with no such risk. Hence, the rationale for this study is not only the need to examine in depth the use of social media in relation to health, but also to use nationally representative data encompassing various individual determinants and health indicators. So far, there has been no such research on Finnish school-aged children. 

This study aimed to examine the association between adolescents’ social media use and health plus health behaviour, using nationally representative Finnish data from the Health Behaviour in School-aged Children (HBSC) survey. The specific research questions for the study were:

- What are the comparative prevalence figures for groups categorized as no-risk (i.e., at no risk for problematic social media use), moderate risk (i.e., at heightened risk of problematic social media use), and problematic in their social media use? (RQ1)
- How are various individual determinants (gender, age, family affluence, parental monitoring, health literacy, academic aspiration, school achievement) associated with social media use? (RQ2)
- How are various social media user groups (no-risk, moderate risk, and problematic use) associated with various health indicators (health complaints, physical inactivity, loneliness, low self-rated health, morning tiredness, short sleep) (RQ3), and, how much does the risk of negative health indicators increase between groups? (RQ4)

## 2. Materials and Methods

### 2.1. Design

Nationally representative data were collected from Finnish adolescents as part of the HBSC study in 2018. Stratification in sampling was based on European Union NUTS classification (Nomenclature of Territorial Units for Statistics). The sampling was based on the Finnish national school register and was carried out via a random cluster sampling method adjusted for the province, municipality, and school size. Probability proportional to size sampling method (PPS) was applied in school selection. The primary sampling unit (PSU) was the school, and within the school the class was randomly selected. Participation was voluntary, and the student response rate was 57%. Anonymous data were collected online by Webropol software (Webropol Oy, Helsinki, Finland). The Ethics committee of the University of Jyväskylä gave approval regarding ethical issues, and the school principals gave school-level approval.

### 2.2. Participants

The HBSC study focuses on collecting the data from 11-, 13-, and 15-year-olds following the international HBSC protocol [14]. The participants were 3408 Finnish adolescents (11 yrs, n = 993; 13 yrs, n = 1246; 15 yrs, n = 1169). The sample included an almost equal number of boys (n = 1706) and girls (n = 1702), and gender and age were not associated (χ^2^(2) = 0.027, *p* = 0.987). 

### 2.3. Measures

Self-reported *gender* and *age* were measured by asking participants to select the correct alternative.

*Problematic social media use* was measured with the 9-item Social Media Disorder Scale (SMD scale) using a dichotomous (No/Yes) answer scale [8]. The items covered preoccupation, tolerance, withdrawal, displacement, escape, problems, deception, displacement, and conflict. Based on the values obtained, the respondents were categorized into three groups: a no-risk group, a moderate risk group (i.e., at a heightened risk of developing problematic use), and a problematic use group. The cut-off value for the problematic use group was 6 or more yes answers; for the moderate risk group the corresponding values were 2 to 5, and for the no-risk group 0 to 1 [38].

*Family affluence* scale III [39] was used to measure the self-reported socioeconomic position, via six items: ownership of a car, having one’s own bedroom, number of family computers, number of bathrooms, ownership of a dishwasher at home, number of family holidays during the past 12 months. The computed scores were recoded into three categories to indicate levels of relative affluence: low family affluence (lowest 20%), medium family affluence (middle 60%), and high family affluence (highest 20%) [40]. *Parental monitoring* was measured by six items, via a 3-point scale focusing on adolescents’ perceptions of parental monitoring and awareness regarding friends, spending money, after-school and free-time activity, internet activity, and going out at night [41]. The responses for monitoring by both mother and father were computed to obtain a sum score that was recoded into three categories: low parental monitoring (lowest 33.3%), medium parental monitoring (middle 33.3%), and high parental monitoring (highest 33.3%).

*Educational aspiration* was measured by a question covering seven educational/vocational tracks after comprehensive school. The response options upper secondary school and double examination (conducted at both upper secondary school and vocational school) were combined and labelled academic plans, while the others were labelled non-academic plans. *Health literacy* was measured by the Health Literacy for School-Aged Children (HLSAC) instrument [42,43]. The scale includes 10 items measuring adolescents’ knowledge and competencies to promote health. The responses were computed to a sum score, which was then recoded into three categories: low health literacy (values 10–25), medium health literacy (values 26–35), and high health literacy (values 36–40) [44]. *Academic achievement* was measured by asking students to indicate their most recent mark in the school subjects first language and mathematics. The mean value for the two marks was calculated and recoded into three categories: low school achievement (mean value 4–7), medium school achievement (mean value 7.5–8.5), and high school achievement (mean value 9–10).

*Health complaints* were measured by the HBSC symptom checklist (HBSC-SCL) [45]. Respondents evaluated the frequency of various complaints, both somatic (headache, neck, and shoulder pain) and psychological (feeling low, nervousness, irritability) over the last six months. The response options about every week, more than once a week, and about every day were combined to indicate frequent and regularly experienced psychosomatic symptoms. Multiple health complaints were defined as experiencing three or more health complaints weekly or more often. *Physical inactivity* was measured using Moderate-to-Vigorous-Physical-Activity (MVPA) questions [46]. Persons meeting the MVPA guidelines on only 0 to 2 days per week were categorized as physically inactive. *Loneliness* was measured by one item: Do you ever feel lonely? The response options very often and quite often were combined to indicate frequently experienced loneliness. *Self-rated health* (SRH) was evaluated by a single question measuring the individual’s perception and evaluation of their health [47]. The response options fair and poor were combined to indicate low SRH.

*Morning tiredness* was measured with a single item: How often do you feel tired when you get up on school mornings? The response options varied from rarely or never to 4 or more times a week. Being tired 4 or more times a week was categorized as a risk for adolescent health. Sleep duration was calculated as the difference between bedtime and wake-up time on school days. Bedtimes were inquired about as follows: When do you usually go to bed if the next morning is a school day? Wake-up times were asked via the question: When do you usually wake-up on school mornings? The response alternatives for both questions were set at half-hour intervals. Sleep duration of at most 7 hours a night was categorized as a risk for adolescent health and referred to as *short sleep*.

### 2.4. Analyses

The descriptive analysis included frequencies, percentages, confidence intervals (CIs), and associations between social media use and background variables (χ^2^ test). 

The associations between social media addiction and health indicators were analysed by binary logistic regression. The analyses were adjusted for PSU. Odds ratio (OR) values were calculated, indicating the strength of the association and the increased risk level. Missing data were handled via a listwise deletion procedure. The significance level for all the analyses was set at *p* < 0.05. All the analyses were conducted by Stata (version 16) (StataCorp LLC, College Station, TX, USA).

## 3. Results

### 3.1. Prevalence of No-Risk, Moderate Risk, and Problematic Social Media Use (RQ1), and Associated Individual Determinants (RQ2)

For the total sample, the problematic social media prevalence was 9.4% and the moderate risk prevalence 33.5% (Table 1). Overall, no gender differences were found regarding problematic use, but girls more than boys belonged to the moderate risk group. Problematic use was more prevalent among 13- and 15-year-old adolescents than among 11-year-olds (11.2% for the 13- and 15-year-olds versus 5.9% for the 11-year-olds). Furthermore, higher parental monitoring and higher health literacy were statistically significantly associated with a lower prevalence of moderate risk and problematic social media use. Low academic achievement was linked to a higher prevalence of problematic social media use. There was no association between family affluence or educational aspiration and the prevalence of no-risk, moderate risk, or problematic social media use. 

### 3.2. Prevalence of Negative Health Indicators among No-Risk, Moderate Risk, and Problematic Users (RQ3)

No-risk users experienced the fewest health complaints or other negative health indicators, and problematic users reported the most. In the total sample, the prevalence of multiple health complaints within the moderate risk group was 33.0%, and in the problematic group 41.4% (Table 2). Overall, among the problematic users the most common negative health indicators were irritability and nervousness.

### 3.3. Associations between Social Media User Groups and Negative Health Indicators (RQ4)

Adolescents in the problematic social media user group were three times more likely, and adolescents in the moderate risk group twice as likely to experience irritability, nervousness, and feeling low. In addition, problematic users were twice as likely to suffer from neck and shoulder pain and headache as compared to the no-risk group. All the OR values for the moderate risk and problematic group were statistically significant at level *p* < 0.001. (Table 3).

The prevalence of morning tiredness, short sleep, and loneliness was over three times as likely, and inactivity twice as likely among the problematic social media users as compared to the no-risk users. Furthermore, loneliness was twice as likely to be experienced by the moderate risk group as compared to the no-risk group (Table 4).

## 4. Discussion

Using a nationally representative sample from Finland, this study sheds new light on adolescents’ social media use and related individual determinants and health indicators. It represents one of the few to offer information on how being a moderate or problematic social media user increases the likelihood of reporting various unfavourable health indicators; hence, it highlights the need to examine not only problematic versus non-problematic users, but also persons at moderate risk of developing problematic social media use.

Problematic social media use is of concern for 9.4% of Finnish adolescents. This is close to the European average (7.4%) [18], while a further 33.5% could be characterized as at moderate risk of developing problematic behaviour. Problematic social media use was most common among the older age groups, and among persons with moderate or low school achievement, low health literacy, and low parental monitoring. Furthermore, belonging to a moderate risk group was most frequent among girls and among persons with low to moderate parental monitoring. Family wealth was not linked to social media use in any of the examined groups. One reason for this may be the inexpensive cost of internet access in Finland, as reported by the European Commission [48], which notes that “Finland has developed an equitable and inclusive information society”.

Our findings did not support recent findings on girls as more likely than boys to report problematic social media use [9,21]. However, a larger proportion of girls (40%) than boys (28%) belonged to the group with a moderate risk for problematic use, indicating a need to pay special attention to girls in attempts to prevent problematic social media behaviour. Research has shown that several social media behaviours, such as selfie behaviour, are typical of both genders, but that for instance appearance concerns explain problematic social media use only among boys [49]. This highlights the need to study in detail the gender differences in social media use and the patterns explaining them. In addition, since this is the first time that Finnish adolescents’ social media use has been measured via a specific problematic-use instrument, we do not know how gender differences have developed over time.

Problematic social media use by adolescents has widely been recognized as a possible indicator for negative health outcomes e.g., [18,21,50]. The findings clearly indicated that among the moderate risk users and the problematic users, there was a higher frequency of various psychosomatic complaints and other negative health indicators than in the no-risk group. For example, among the adolescents identified as belonging to the no-risk group, 15% reported having multiple health complaints and 36% nervousness on a weekly or daily basis. In the moderate risk group, the corresponding figures were 33% (multiple health complaints) and 59% (nervousness), and in the problematic use group 41% (multiple health complaints) and 66% (nervousness). Furthermore, the prevalence of weekly morning tiredness increased from 25% (no-risk group) to 56% (problematic use group), and short sleep from 18% to 39%. One alarming finding was that the risk of loneliness was double for the moderate risk group and triple for the problematic use group. There have been mixed findings on the effects of social media on loneliness, and the differences have been explained via the individual’s orientation to social comparison (such that persons with a higher orientation towards social comparison have an increased risk of social media increasing loneliness) [51].

Given that the risk for all the measured negative health indicators systematically increased if the respondent belonged to a moderate risk or a problematic use group, there is a need to broaden the scope of investigation to persons at moderate risk of developing problematic behaviour instead of studying only problematic versus no-risk users. We found an increased risk for 9 out of 11 measured unfavourable health indicators among the moderate risk group, and often the risk was over twice as large as that for the no-risk group. Moreover, in relation to 5 out of 11 measured unfavourable health indicators (headache, feeling low, nervousness, irritability, and having multiple complaints), the risk increased if the respondent belonged to the moderate risk group as compared to the no-risk group, with no detectable difference between moderate risk and problematic users. In addition, given that 43% of the Finnish adolescents belonged to either the moderate risk (34%) or the problematic group (9%), with only small age differences, the magnitude of the findings on the associations between social media use and health should not be underestimated. As Clark et al. [6] have argued, “the health and societal costs of problematic use of the internet across the lifespan are unknown, but they could be huge”. The critical question is what “moderate risk” actually means. In a positive sense, one could say that at this stage adolescents still have control over their social media use. However, it is difficult to say how near persons in this group are to developing a problematic behaviour.

The cross-sectional data do not allow causal inferences and arguments on whether various negative health outcomes predispose towards or are consequences of problematic social media use [5]. In addition, in this study we examined the associations between problematic social media use and health indicators but did not assess the different types of social media used by the participants. Hence, caution is needed in generalizing from the results, given that the associations of different types of media can vary greatly. One should also note the role of probable mediating factors between social media use and health. For instance, Viner et al. [52] found that a lack of sleep or of physical activity mediated the association between social media use and various mental health problems, and they considered direct causalities between extensive social media use and health problems to be very unlikely. However, in an experimental study among university students conducted by Hunt et al. [53] it was observed that limitations on social media use may decrease loneliness and depressive symptoms. Heffer et al. [54] took the view that that social media use did not in itself predict mental health problems; in fact, causality operated the other way round. Undoubtedly, further research is needed to avoid a situation in which various health problems are explained purely via social media use, with technology regarded as “an illness that society must address” [3]. According to Boyd [3], “It is easier for adults to blame technology for undesirable outcomes than to consider other social, cultural, and personal factors that may be at play.” More broadly, research is needed to explore problematic social media use and its associations with health in a more comprehensive manner, using for instance an ecological approach to identify explanatory factors across several domains (i.e., individual, family, peer, school and community) in the same sample, and at the same time.

## 5. Conclusions

Based on the findings of this study, more research is needed on the role of health literacy and school achievement in empowering adolescents to have control over their social media use, with attention given also to the role of parental monitoring in creating a supportive environment for more controllable social media use. However, one can suggest that preventative interventions should be comprehensive in nature, i.e., they should focus on individuals and their environments, and be targeted at the whole population, albeit with a special focus on those at moderate risk and those with problematic habits. One must accept that the internet and social media play a significant role in people’s lives, and that prohibitions of it are practically impossible [55]. Hence, interventions should focus on factors that support adolescents’ skills in regulating their own behaviour [56].

## Figures and Tables

**Table 1:** The prevalence of no-risk, moderate risk, and problematic social media user groups: totals, and proportions by gender, age, family affluence, parental monitoring, educational aspiration, health literacy, and academic achievement.

Variable	No-Risk Social Media Use	Moderate Social Media Use	Problematic Social Media Use	Total	χ^2^(df); *p*-Value
	% (95% CI)	% (95% CI)	% (95% CI)	% (n)	
All	57.1 (54.8–59.3)	33.5 (31.6–35.5)	9.4 (8.3–10.8)	100 (3077)	
Gender					χ^2^(2) = 76.98; <0.001
Boys	65.1 (62.2–67.8)	26.6 (24.1–29.3)	8.3 (6.9–10.1)	100 (1496)	
Girls	49.6 (46.5–52.7)	40.0 (37.2–42.8)	10.5 (8.8–12.4)	100 (1581)	
Age					χ^2^(4) = 27.06; <0.001
11 years	60.5 (56.7–64.2)	33.6 (30.3–37.2)	5.9 (4.5–7.6)	100 (912)	
13 years	53.5 (49.6–57.4)	35.3 (31.6–39.1)	11.2 (9.0–14.0)	100 (1122)	
15 years	57.1 (53.5–60.6)	31.7 (28.9–34.7)	11.2 (9.2–13.5)	100 (1043)	
FAS					χ^2^(4) = 4.89; <0.353
Low	57.3 (53.0–61.4)	32.2 (28.4–36.1)	10.6 (8.3–13.4)	100 (663)	
Medium	56.1 (53.2–58.9)	34.8 (32.4–37.2)	9.2 (7.7–10.8)	100 (1782)	
High	60.2 (55.7–64.5)	31.6 (27.4–36.0)	8.2 (6.1–11.0)	100 (552)	
Parental monitoring					χ^2^(4) = 93.30; <0.001
Low	46.1 (41.8–50.4)	37.3 (33.6–41.1)	16.7 (13.8–19.9)	100 (644)	
Moderate	51.4 (46.9–55.9)	38.9 (34.5–43.5)	9.7 (7.6–12.1)	100 (669)	
High	68.7 (64.9–72.3)	25.5 (22.0–29.4)	5.8 (4.1–8.0)	100 (681)	
Educational aspiration					χ^2^(2) = 16.66; <0.080
High school	58.3 (54.3–62.3)	32.3 (29.0–35.9)	9.4 (7.2–12.1)	100 (700)	
Vocational	55.2 (49.3–61.1)	30.6 (25.7–36.0)	14.2(10.7–18.6)	100 (321)	
Health literacy					χ^2^(4) = 94.80; <0.001
Low	42.8 (33.9–52.2)	34.7 (27.0–43.3)	22.4 (15.8–30.8)	100 (180)	
Moderate	50.4 (47.3–53.5)	38.2 (35.1–43.3)	11.2 (9.2–14.0)	100 (1175)	
High	65.2 (61.2–68.9)	25.9 (22.7–29.4)	9.0 (7.0–11.4)	100 (739)	
Academic achievement					χ^2^(4) = 54.74; <0.001
Low	53.2 (47.8–58.5)	30.3 (26.1–34.8)	16.6 (12.9–20.9)	100 (535)	
Moderate	55.2 (51.5–59.0)	33.0 (29.5–36.7)	11.8 (10.0–14.3)	100 (970)	
High	57.7 (52.8–62.5)	37.0 (32.4–41.7)	5.4 (3.7–7.9)	100 (585)	

**Table 2:** Prevalence of negative health indicators among no-risk, moderate risk, and problematic user groups.

Health Indicators	No-Risk Social Media Use	Moderate Risk Social Media Use	Problematic Social Media Use	χ^2^(df); *p*-Value
	% (95% CI)	% (95% CI)	% (95% CI)	
Health complaints ^a^				
Headache	32.0 (29.5–34.6)	46.3 (42.7–49.8)	51.9 (46.0–57.8)	χ^2^(2) = 79.4; <0.001
Neck & shoulder pain	26.4 (24.1–28.9)	37.4(34.1–40.9)	47.5 (41.2–53.9)	χ^2^(2) = 71.1; <0.001
Feeling low	26.1 (23.6–28.8)	49.2 (45.5–52.9)	53.5 (47.2–59.6)	χ^2^(2) = 188.4; <0.001
Nervousness	36.4 (33.8–39.1)	59.4 (55.7–62.9)	65.6 (59.7–71.0)	χ^2^(4) = 182.4; <0.001
Irritability	42.4 (39.6–45.1)	64.8 (61.4–68.1)	70.7 (65.1–75.8)	χ^2^(2) = 173.4; <0.001
Multiple health complaints	14.6 (12.8–16.6)	33.0 (29.7–36.6)	41.4 (35.3–47.9)	χ^2^(2) = 181.8; <0.001
Other health indicators ^b^				
Physical inactivity	10.2 (8.5–12.2)	12.1 (10.1–14.8)	23.4 (19.1–28.3)	χ^2^(2) = 40.6; <0.001
Loneliness	9.2 (7.7–10.9)	18.4 (15.9–21.2)	27.4 (22.2–33.2)	χ^2^(4) = 93.6; <0.001
Low SRH	12.6 (10.9–14.6)	18.8 (16.2–21.7)	29.7 (24.3–358)	χ^2^(2) = 60.1; <0.001
Morning tiredness	25.3 (23.0–27.7)	38.0(34.7–41.4)	55.9 (49.3–61.8)	χ^2^(2) = 126.2; <0.001
Short sleep	17.7 (15.2–20.5)	23.9 (20.4–28.0)	39.3 (32.9–46.0)	χ^2^(2) = 55.7; <0.001

Note: ^a^ percentage of respondents in each social media use group reporting health complaints weekly or more often. ^b^ percentage of respondents in each social media use group reporting other negative health indicators.

**Table 3:** Binary logistic regression on the associations between social media user groups and psychosomatic health complaints.

Groups	Health Complaints					
	Headache	Neck and Shoulder Pain	Feeling Low	Nervousness	Irritability	Multiple Health Complaints
	OR (95% CI)	OR (95% CI)	OR (95% CI)	OR (95% CI)	OR (95% CI)	OR (95% CI)
Addiction group						
No-risk	1	1	1	1	1	1
Moderate risk	1.68 (1.40–2.02) ***	1.57 (1.30–1.89) ***	2.46 (2.05–2.96) ***	2.35 (1.96–2.81) ***	2.31 (1.95–2.73) ***	2.59 (2.10–3.20) ***
Problematic	2.13 (1.65–2.75) ***	2.35 (1.76–3.12) ***	2.94 (2.22–3.88) ***	3.11 (2.37–4.07) ***	3.05 (2.34–3.98) ***	3.77 (2.77–5.13) ***
Gender						
Boy	1	1	1	1	1	1
Girl	1.76 (1.47–2.10) ***	1.54(1.30–1.83) ***	2.71 (2.25–3.26) ***	1.79 (1.51–2.11) ***	1.86 (1.59–2.17) ***	2.52 (2.06–3.07) ***
Age						
11 years	1	1	1	1	1	1
13 years	1.09 (0.85‒1.39)	1.11 (0.87–1.40)	1.19 (0.91–1.56)	1.19 (0.94–1.50)	1.14 (0.89–1.46)	1.12 (.85–1.47)
15 years	1.25 (1.01–1.54) *	1.48 (1.19–1.84) **	1.73 (1.34–2.21) ***	1.11 (0.89–1.39)	1.25 (1.00–1.57)	1.65 (1.27–2.15) ***

Note: * *p* < 0.05, ** *p* < 0.01, *** *p* < 0.001.

**Table 4:** Binary logistic regression on the associations between social media user groups and other negative health indicators.

Groups	Health Indicators				
	Physical Inactivity	Loneliness	Low SRH	Morning Tiredness	Short Sleep
	OR (95% CI)	OR (95% CI)	OR (95% CI)	OR (95% CI)	OR (95% CI)
Addiction group					
No-risk	1	1	1	1	1
Moderate risk	1.25 (0.96–1.65)	2.05 (1.62–2.59) ***	1.60 (1.28–1.99) ***	1.76 (1.47–2.09) ***	1.60 (1.25–2.06) ***
Problematic	2.46 (1.81–3.35) ***	3.37 (2.42–4.70) ***	2.68 (1.99–3.61) ***	3.43 (2.61–4.51) ***	3.23 (2.32–4.50) ***
Gender					
Boy	1	1	1	1	1
Girl	1.00 (0.77–1.31)	1.94(1.54–2.46) ***	1.02 (0.82–1.26)	1.26 (1.07–1.50) **	0.73 (0.59–0.92) **
Age					
11 years	1	1	1	1	NA
13 years	2.11 (1.43–3.11) ***	1.18 (0.85–1.64)	2.12 (1.54–2.93) ***	1.44 (1.16–1.78) **	1
15 years	4.84 (3.42–6.85) ***	1.95 (1.43–2.65) ***	2.31 (1.64–3.26) ***	1.85 (1.51–2.27) ***	1.60 (1.22–2.09) **

Note: ** *p* < 0.01, *** *p* < 0.001.

## Data Availability

The data presented in this study are available on reasonable request from the corresponding author. The data are not publicly available due to ethical requirements.
